# Clinicopathological characteristics of cancer associated with Crohn’s disease

**DOI:** 10.1007/s00595-016-1336-2

**Published:** 2016-04-19

**Authors:** Hirofumi Sasaki, Hiroki Ikeuchi, Toshihiro Bando, Kei Hirose, Akihiro Hirata, Teruhiro Chohno, Yuki Horio, Naohiro Tomita, Seiichi Hirota, Yoshihiro Ide, Yasuaki Tsuchida, Motoi Uchino

**Affiliations:** 1 Departments of Inflammatory Bowel Disease, Hyogo College of Medicine, 1-1 Mukogawa-cho, Nishinomiya, Hyogo 663-8501 Japan; 2 Departments of Surgery, Hyogo College of Medicine, Nishinomiya, Japan; 3 Departments of Surgical Pathology, Hyogo College of Medicine, Nishinomiya, Japan

**Keywords:** Crohn’s disease, Cancer, Anorectal lesion, Prognosis

## Abstract

**Purpose:**

We examined the clinicopathological characteristics and prognosis of patients with cancer associated with Crohn’s disease (CD).

**Methods:**

The subjects of this study were patients with cancer confirmed in a resected specimen of bowel, who were treated at our institution between September, 1974 and December, 2014.

**Results:**

We analyzed 34 patients (26 men, 8 women, median age at cancer diagnosis 43.5 years, duration of illness 18 years) and found that the number of those with CD complicated with cancer began to drastically increase after 2005. The site of onset of cancer was in an anorectal lesion in 24 (70.6 %) patients. In 17 (50 %) patients, the cancer was diagnosed before surgery; in 3 patients (8.8 %), it was based on pathological findings during surgery; and in 14 patients (41.2 %), it was based on postoperative pathological findings. Mucinous carcinoma was the dominant histological type, seen in 15 patients (44.1 %), while the special type of signet-ring cell carcinoma was found in 4 patients. The cumulative overall 5 year survival rate was 46.2 %.

**Conclusion:**

In this group of Japanese CD patients, an anorectal lesion was the most frequent site of origin of cancer. As cancer was diagnosed preoperatively in only 50 % of these patients, the overall prognosis was poor, with a cumulative 5 year survival rate of just 46.2 %.

## Introduction

The association of cancer with CD was not known until many years after the classic description of “regional enteritis” in 1932; thus, information about cancer epidemiology in those patients is limited. Recent studies have found that CD is associated with an increased risk of malignancy [1, 2]. Three types of gastrointestinal carcinomas have been shown to occur more frequently in patients with CD than in the general population: small bowel cancer, colorectal cancer (CRC), and carcinoma arising from a perianal fistula [2–6], with the presence of anal carcinoma in these patients clearly associated with a long-standing perianal fistula. It is important to note that malignancy can arise in association with CD-related perianal disease. Thus, attending physicians should have a high level of suspicion towards cancer in patients with long-standing perianal CD who report a change in symptoms.

## Patients and methods

### Patients

Between September, 1974 and December, 2014, 1096 patients underwent surgery for CD at our institution. Cancer was confirmed in specimens resected from 34 of these patients, who were the subjects of this study.

## Methods

We analyzed retrospectively the clinicopathological characteristics and prognoses of patients with cancer associated with CD, who were treated at our institution. The cancers were staged according to the 7th UICC TNM staging system based on results of the final pathological examination, and follow-up examinations were performed until the time of death or closing date of the study (March 31, 2015).

### Statistical analysis

All statistical analyses were performed using JMP version 11 (SAS Institute Inc., Cary, NC). Fisher’s exact test was used for categorical data. Otherwise, patients were divided into two groups based on the median value of their age at onset and at their first operation (24- and 37-years-old, respectively). Multivariate analysis findings are expressed as odds ratios and 95 % confidence interval values. A *p* value of <0.05 was considered significant. Cumulative 5 year survival was analyzed using the Kaplan–Meier method and the log-rank test.

## Results

### Changes in the number of cancer cases

**Fig. 1:**
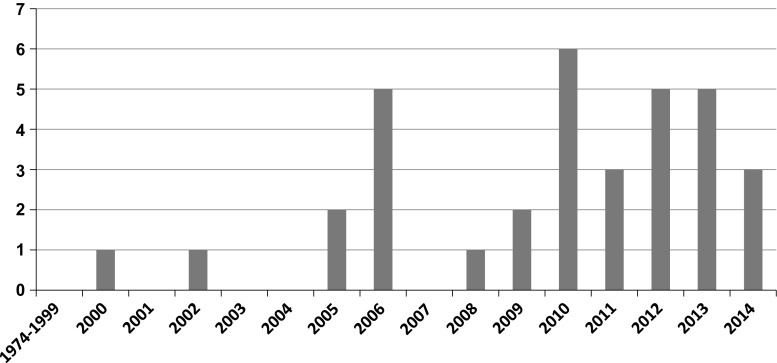
Changes in the number of cases of colorectal cancer among patients with Crohn’s disease (CD). The number of CD patients with cancer increased dramatically after 2005

In 2000, we encountered our first case of cancer associated CD and from 2005, the number of cases of CD complicated by cancer increased dramatically. By December, 2014, we had treated 34 such patients in our department (Fig. 1).

### Clinical characteristics

There were 26 men and 8 women, with a median age at cancer diagnosis of 43.5 years and a duration of illness of 18 years (Table 1).

**Table 1:** Clinical characteristics

Sex (male/female)	26/8
Age at onset in years (range)	24.0 (11–60)
Age at cancer diagnosis in years (range)	43.5 (27–73)
Duration of disease in years (range)	18 (6–37)
Extent of disease at first operation	Ilitis: 4, colitis: 2, ileocolitis: 28

### Location of CD-related cancer

Table 2 shows the sites where cancer arose in our patients with CD. The lesions were most commonly found in the anal fistula (*n* = 9, 26.5 %), followed by the anal canal (*n* = 8, 23.5 %), and rectum (*n* = 7, 20.6 %). CD-related cancer was discovered in anorectal lesions in 24 (70.6 %) of our patients.

**Table 2:** Location of cancer in CD patients (*n* = 34)

Location	Patients (%)
Fistula	4 (11.8)
Entero-enteric	3 (8.8)
Entero-cutaneous	1 (2.9)
Intestine	30 (88.2)
Ileum	2 (5.9)
Cecum	2 (5.9)
Descending colon	1 (2.9)
Sigmoid colon	1 (2.9)
Rectum	7 (20.6)
Anal fistula	9 (26.5)
Anal canal	8 (23.5)

### Time and method of diagnosis

Table 3 shows the time and methods of diagnosis. In 17 patients (50 %), the diagnosis was made before surgery, whereas in 3 patients (8.8 %) it was based on pathological findings during surgery and in 14 (41.2 %), it was based on postoperative pathological findings.

**Table 3:** Time and method of diagnosis

Time	Method	No. of cases
Preoperative	Endoscopy	12
	Curettage of fistula	3
	Direct tumor biopsy	2
Intraoperative	Pathological examination	3
Postoperative	Pathological examination	14

### Histology

Mucinous carcinoma was the dominant type of cancer, diagnosed in 15 patients (44.1 %), while special type signet-ring cell carcinoma was diagnosed in 4 (Table 4).

**Table 4:** Histology

Location	Histology	No. of cases
Fistula	Poorly differentiated	1
	Signet-ring cell	1
	Mucinous	1
	Squamous cell	1
Ileum	Well differentiated	1
	Poorly differentiated	1
Colon	Well differentiated	3
	Mucinous	1
Anal-rectal lesion	Mucinous	13
	Well differentiated	5
	Moderately differentiated	3
	Signet-ring cell	3

### Progression, stage, and prognosis

Table 5 shows the progression, stage, and prognosis on the closing date of the study. The mean period until death was 14.5 months (range 3–109 months). For the patients who survived, the mean period from operation to the closing date of the study (March 31, 2015) was 35.3 months (10–129 months). The stage was analyzed after excluding special cases such as fistula cancer. Stage II was the most common, found in 16 patients, 8 of whom were recurrence-free, 2 of whom had local recurrence, and 1 of whom had distal metastasis. Five patients with recurrence died of the disease.

**Table 5:** Progression stage and prognosis

Stage	Prognosis	No. of cases
0, I (6)	No recurrence	5
	Local recurrence	1
II (16)	No recurrence	8
	Local recurrence	2
	Distance metastasis	1
	Death	5
IIIa (2)	Death	2
IIIb (4)	No recurrence	2
	Death	2
IIIc (l)	Death	1
IV (1)	Death	1
Classification not possible (4)	Death	4

### Preoperative treatment and cancer remaining after surgery (Table 6)

Eight of the 24 CD patients with cancer discovered in an anorectal lesion underwent chemoradiotherapy. Twenty (58.8 %) of the 34 patients in the total cohort underwent microscopic curative resection and 14 (41.2 %) underwent macroscopic or microscopic non-curative resection, or the remaining cancer could not be detected.

**Table 6:** Preoperative treatment and cancer remaining after surgery

Location	No. of cases	Preoperative CRT	Level of remaining cancer			
			RX	R0	R1	R2
Fistula	4	0	2	2		
Entero-enteric	3	0	1	2		
Entero-cutaneous	1	0	1			
Intestine	30		2	18	7	3
Ileum	3	0		2		1
Colon	3	0		3		
Rectum	7	2		5	2	
Anal fistula	9	4	2	4	2	1
Anal canal	8	2		4	3	1

*CRT* chemo-radio therapy, *RX* remaining cancer could not be determined, *R0* no cancerous remnant, *R1* resected surface of remnant histologically positive, *R2* large portion of cancerous tissue remained

### Cumulative 5 year survival rate

**Fig. 2:**
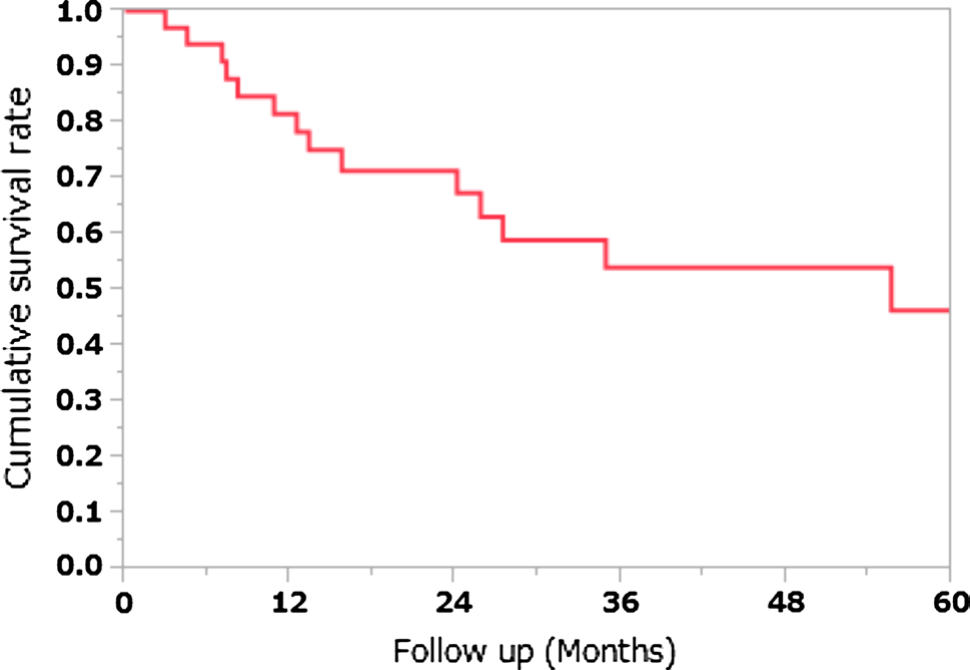
Cumulative 5-year survival rate. The cumulative 5 year survival rate was 46.4 %

Follow-up examinations were performed until either death or the closing date of the study (March 31, 2015). The cumulative overall 5 year survival rate was 46.2 % (Fig. 2).

### Pathological types and cumulative 5 year survival rate

**Fig. 3:**
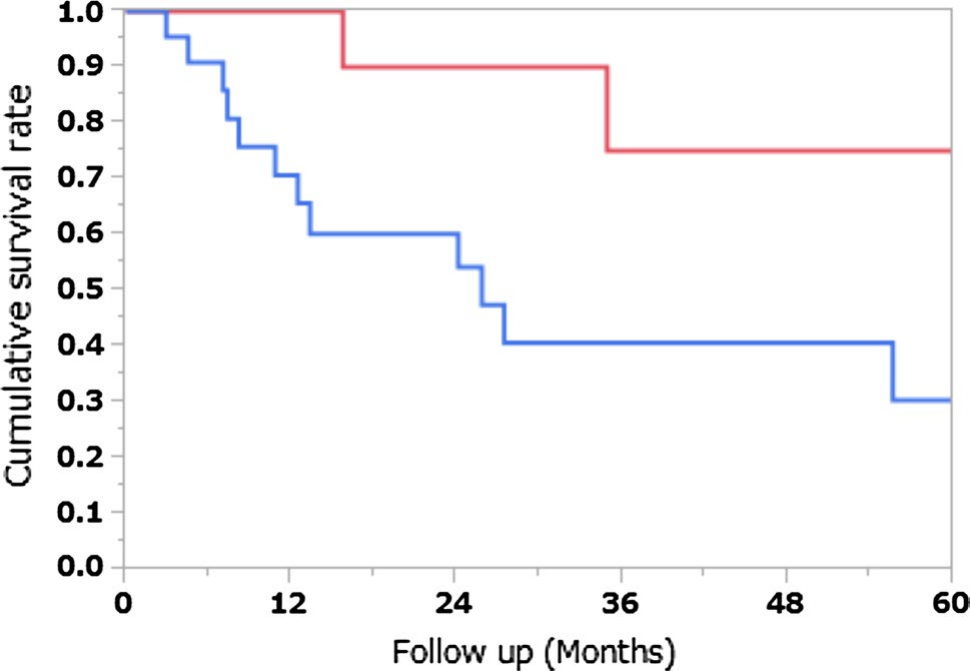
Pathological types and cumulative 5 year survival rate

The cumulative 5 year survival rate for the patients with moderately differentiated or well differentiated adenocarcinoma was 75.0 %, whereas that for the patients with other histological types (poorly differentiated adenocarcinoma, mucinous carcinoma, signet-ring cell carcinoma, or squamous cell carcinoma) was significantly worse at 30.5 % (*p* = 0.02; Fig. 3).

### Risk factors for cancer development in CD patients

Table 7 details the results of univariate and multivariate analyses. Univariate analysis showed a significant difference in the site of disease at the first operation and age at the first operation. The presence of an anal lesion at the first operation and smoking status were not shown to be significant factors. These results were confirmed by multivariate analysis, which revealed extent of disease and age at the first operation as significant factors.

**Table 7:** Univariate and multivariate analyses of risk factors for cancer associated CD

Factors	Univariable analysis				Multivariable analysis		
	Cancer	OR	95 % CI	*P*	OR	95 % CI	*P*
Age at onset							
≥24 years	17 (50.0)	1.24	0.62–2.46	0.600			
<24 years	17 (50.0)						
Sex							
Man	26 (76.5)	0.81	0.36–1.81	0.699			
Woman	8 (23.5)						
Age at first operation							
≥37 years	17 (50.0)	3.00	1.51–5.97	0.0022	3.38	1.68–6.79	0.0008
<37 years	17 (50.0)						
Site of disease							
Ilitis	5 (14.3)	3.45	1.20–9.88	0.0134	3.99	1.54–6.79	0.0027
Ileo-colitis + colitis	29 (85.3)						
Anal lesion at initial surgery							
Present	23 (67.6)	1.59	0.76–3.29	0.223			
Absent	11 (32.4)						
Type of disease							
Perforating	18 (52.9)	1.52	0.77–3.02	0 221			
Non-perforating	16 (47.1)						
Active smoking							
Yes	7 (20.6)	0.63	0.27–1.47	0.338			
No	27 (79.4)						
Alcohol habit							
Yes	4 (11.8)	0.79	0.27–2.29	0.179			
No	30 (88.2)						

## Discussion

Colorectal cancer (CRC) in a patient with CD was first described in 1948 [7]. Recent studies have demonstrated that CD carries an increased risk of malignancy [1, 2], although it should be noted that there are regional differences in regard to the location of cancer in those patients. Stahl et al. reported that only 17 % (*n* = 4) of cancerous lesions in their CD patients were located in the rectum, which was a much lower incidence than for sporadic cancer (38 %). On the other hand, 59 % (*n* = 14) were located in the ascending or transverse colon, which was significantly higher than for sporadic cancer (28 %) [8]. Moreover, Kersting et al. reviewed previous studies and found that 39–50 % of CD-associated tumors were located in the rectum [5, 9]. They noted that the majority of CD-associated tumors were located in an intestinal segment that was easily accessible for an endoscopic examination. On the other hand, Mizushima et al. reported that 34 of 44 CRCs (77.3 %) in Japanese patients with CD arose in the sigmoid colon, rectum, or anal canal/fistula [10]. According to some reports from Western countries, patients with CD showed an increased risk of colon cancer but not rectal cancer [11]. Thus, there may be genetic and environmental factors associated with the development of CRC in patients with CD.

Some studies have investigated the risk of cancer development in CD patients. Scaringi et al. reported that CD patients who require surgery are at higher risk of the development of colorectal cancer, particularly those whose disease duration is >10 years, have distal localization, are <40-years-old at diagnosis, and have penetrating disease [12]. In the present study, the site of disease (colon involvement) at the first operation and age at the first operation were associated with an increased risk of the development of cancer in Japanese CD patients.

**Fig. 4:**
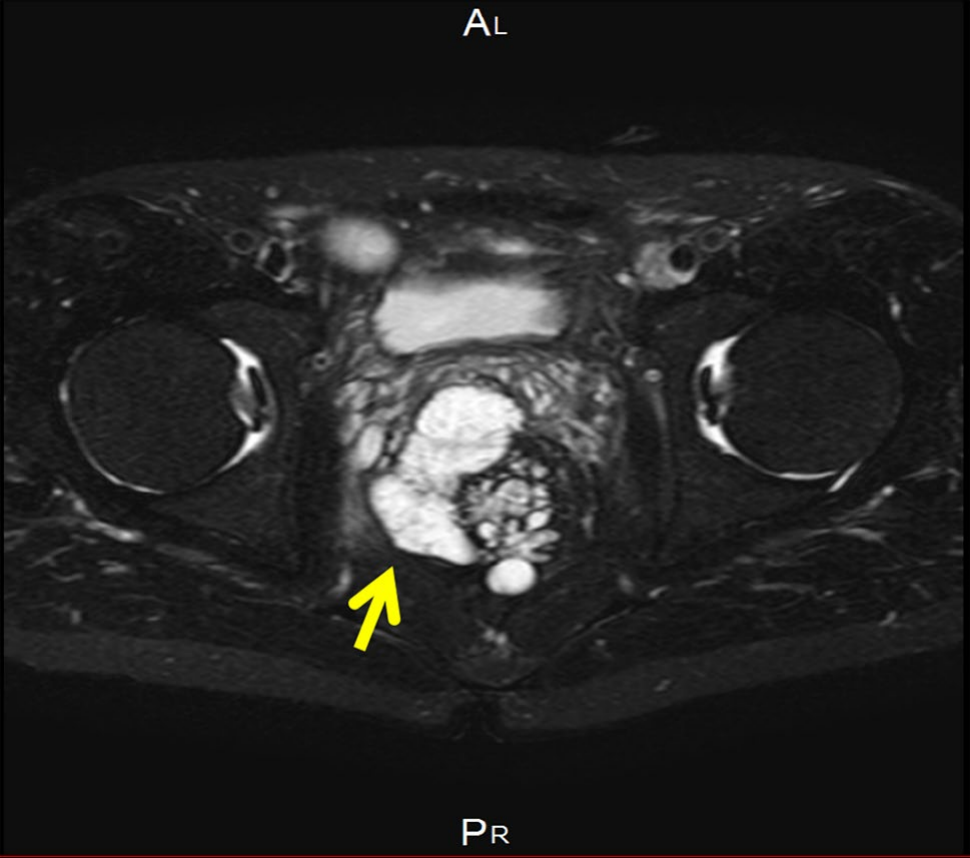
T2-weighted MRI findings of a representative case of mucinous cancer.* Arrow* indicates multilocular cyst

Recent studies have indicated the potential of FDG-PET to assess CD activity [13–15], although to our knowledge, there is no report on its potential to assess CD-associated malignant tumors. We performed FDG-PET in four of our patients prior to surgery and the results were positive in two patients and negative in two [16]. Based on our limited experience, FDG-PET does not have a high accuracy rate, possibly because of the high incidence of mucinous cell type carcinoma among CD-associated cancers. Whiteford et al. also reported that the sensitivity of FDG-PET for detecting mucinous carcinoma was lower than that for non-mucinous cancer [17]. We think that magnetic resonance imaging (MRI) is more effective than FDG-PET for CD patients with anorectal carcinoma. For anorectal cancer, pelvic MRI is the best method for examining cancer localization and invasion, as well as its positional relationship with surrounding organs. An anal fistula carcinoma associated with mucinous cancer frequently shows mucus retention; thus, multilocular cyst-like findings are often observed on T2-weighted MRI images. Figure 4 shows representative T2-weighted MRI findings of a CD patient with mucinous cancer case (multilocular cyst indicated by arrow).

No conclusions can be drawn from the present study about the efficacy of adjuvant chemo-radiation treatment for CD patients with a fistula-associated anal adenocarcinoma because of the small number of subjects analyzed and the retrospective nature of the study. Furthermore, it is difficult to evaluate tumor regression grade in patients with CD-associated lower rectal carcinoma, as most colonic carcinomas in CD patients are of the mucinous cell type. Sengul et al. compared outcomes following preoperative chemo-radiation treatment in 16 patients with mucinous rectal cancer and 55 with non-mucinous rectal cancer [18]. Those with mucinous tumors had significantly more advanced T stage after chemoradiotherapy, whereas only 18 % of those patients had a shift toward earlier T and N stages, compared with 74 % of patients with non-mucinous cancer. On the other hand, Wolfgang et al. reported good results, with combined judging of their results, for all seven of their patients for whom this treatment achieved a complete response. They concluded that neo-adjuvant chemoradiotherapy may play an important role in the treatment of locally advanced disease [19]. In the present study, eight patients were treated with neo-adjuvant chemo-radiation, but by the end of the study period, six of these patients had died, one distant metastasis, and one had local recurrence. Based on these results, we do not consider that neo-adjuvant chemoradiotherapy is effective for patients with CD-associated anorectal cancer.

Although the outcome of patients with a CD-associated cancer is the same as that of those with sporadic cancer at a corresponding stage, the prognosis for the former is often worse due to the advanced stage at time of diagnosis, as there are no formal guidelines for the screening and surveillance of cancer associated with CD. We usually perform a surveillance colonoscopy examination for ulcerative colitis (UC) patients, but colonoscopy is not possible in CD patients because of the associated anorectal stenosis or pain.

We previously reported that the 5 year survival rate of patients with UC-associated colorectal cancer was 89 % [20], which was improved from earlier reports, probably because surveillance colonoscopy is effective for detecting colorectal cancer at an early stage. On the other hand, we do not know of any report on the 5 year survival rate of patients with CD-associated intestinal cancer. In the present study, the cumulative 5 year survival rate for CD-associated cancer was only 46.2 %, which is worse than that for UC patients. Lack of suspicion in the early stages, and inadequate physical or colonoscopy examinations due to exacerbation of perianal symptoms may delay diagnosis. Laurent et al. noted that a high degree of suspicion for carcinoma is needed during rectal examination of CD patients and recommended biopsy, curettage, or brushing of the fistulous tract [21]. We agree that repeated biopsy procedures are necessary and should be performed when there is exacerbation of local symptoms and signs, such as increased pain with discharge or bleeding, or if the attending physician has any concerns.

## Conclusion

Since 2005, the incidence of CD-associated cancer has been increasing in our Japanese patients, with an anorectal lesion the most frequent site of occurrence. An accurate preoperative diagnosis was made in only 50 % of the patients in this series. Thus, patient prognosis was poor, with a cumulative 5 year survival rate of only 46.2 %.
